# Inter- and Intradigit Somatotopic Map of High-Frequency Vibration Stimulations in Human Primary Somatosensory Cortex

**DOI:** 10.1097/MD.0000000000003714

**Published:** 2016-05-20

**Authors:** Mi-Hyun Choi, Sung-Phil Kim, Hyung-Sik Kim, Soon-Cheol Chung

**Affiliations:** From the Department of Biomedical Engineering, Research Institute of Biomedical Engineering, College of Biomedical & Health Science, Konkuk University, Chungju (M-HC, H-SK, S-CC); and Department of Human and Systems Engineering, Ulsan National Institute of Science and Technology, Ulsan (S-PK), South Korea.

## Abstract

Although more about the somatotopic mapping of fingers continues to be uncovered, there is lack of mapping attempts regarding the integration of within-finger and across-finger somatotopic coordinates in Broadmann area (BA) 3. This study aimed to address the issue by finding an inter-/intradigit somatotopic map with high-frequency (250 Hz) vibrotactile stimulation. Functional magnetic resonance imaging (fMRI) data were acquired while stimulation was applied to 3 phalanxes (distal [p1], intermediate [p2], and proximal [p3] phalanx) of 4 fingers (index, middle, ring, and little finger) for a total of 12 finger–phalanx combinations for a human. Inter-, intra-, and inter-/intradigit distances were calculated from peak activation coordinates in BA 3 for each combination. With regard to interdigit dimensions, the somatotopic coordinates proceeded in the lateral-to-medial direction for the index, middle, ring, and little fingers consecutively. This trend is comparable to that generated from low-frequency stimulation modalities (flutter stimulation). The somatotopic distances between fingers were greatest when p1 was compared across fingers. From an intradigit perspective, stimulation on p1, p2, and p3 yielded BA 3 peak coordinates aligned along the anterior-to-posterior and inferior-to-superior directions for all fingers. An inter-/intradigit map exhibited a radially propagating trend of distances calculated with respect to index p1 as a reference point; this provided an integrated view of inter- and intradigit somatotopies, which are traditionally discussed separately. We expect such an inter-/intradigit somatotopic map approach to contribute in generating a comprehensive somatotopic model of fingers.

## INTRODUCTION

Growing developments in haptic interface applications have fostered much attention to research on tactile perception and its correlated brain functions. One of the fundamental fields of this line of research is somatotopic mapping of the hand, which is known to occupy the greatest area in the somatosensory cortex. Especially, the functional mapping of individual fingers and its phalanxes on the cortex is of great interest. With advances in neuroimaging technology such as functional magnetic resonance imaging (fMRI), research is not only confined to traditional animal studies but is also being conducted on human subjects. Many studies have focused on neural activity in the primary somatosensory cortex (S1) evoked by tactile stimulation.^1–8^ The domain of S1 consists of Brodmann area (BA) 1, 2, and 3. In particular, BA 3 should be referred to as a “primary” somatosensory cortical area because it directly receives the bulk of the thalamocortical projections from the sensory input fields.^9–11^ Hence, it has been the subject of numerous studies on somatotopic mapping.^3,4,6,12–17^

Electrophysiological studies on nonhuman primates have provided basic information regarding somatotopic maps of tactile perception.^18–25^ Some groups have reported an interdigit map in BA 3 that proceeds from the inferior to superior areas in the order of the thumb to the little finger.^19,23–25^ An intradigit somatotopic map of first to third phalanxes within a finger has been reported to be arranged in the direction from the anterior to posterior areas in S1.^18–19,21,22^

More recently, finger somatotopy studies on humans have been conducted with various stimulation modalities: piezoelectric (<50 Hz), brush, electric, and air stimulations.^3,4,6,12–17^ Most interdigit studies investigated somatotopic maps in BA 3 by applying various stimulations on the first phalanx of different fingers. Intradigit studies have also employed different types of stimuli to find somatotopic maps. For example, intradigit somatopic mapping studies used electric stimulation on each phalanx of the middle finger,^12^ air stimulation interspaced at 1.5-cm intervals on the thumb, index, and ring fingers,^14^ or piezoelectric stimulation of about 30 Hz (flutter) on the first phalanx and the interconnection between the palm and the index and little fingers.^15,26^ In addition, a study has reported an intradigit somatotopic map based on stimulating each phalanx of the index finger with a 150 Hz vibration stimulus.^27^ Recently, Schweisfurth et al^28^ created an intradigit somatotopic map by providing a 32 Hz flutter stimulus to each of 3 phalanxes in all 5 fingers and measured the intradigit distances in BA 3. Interdigit studies have mostly been limited to mapping of the first phalanx; thus, the somatotopic map of the rest of the phalanxes is still unknown. To the best of our knowledge, there have been few reports on the intradigit distance in the somatotopic distribution of BA 3 that considers the whole map of all finger–phalanx combinations. Motivated by such deficiencies, our intent in this study was to fill in the blank by observing a comprehensive inter-/intradigit somatotopic map.

Most previous studies provided flutter stimulation to fingers to find somatotopic maps in BA 3. Flutter stimulation (<50 Hz) preferentially activates tactile information transmission through rapidly adapting (RA) and slowly adapting (SA) type 1 afferents.^29,30^ RA and SA 1 have a relatively large field density (approximately 140 units/cm^2^) and small receptive field (3–4 mm) and thus demonstrate higher sensitivity.^29,30^ Such properties allow flutter stimulation to be used for developing somatotopic maps of BA 3.^3,4,6,13,15,17,26^. In contrast, reports on somatotopic maps in BA 3 for high-frequency vibration are relatively rare. High-frequency vibration preferentially involves more Pacinian than Meissner afferents. The Pacinian corpuscle possesses a relatively low field density (approximately 25 units/cm^2^) and wide receptive fields compared with the Meissner corpuscle.^29,30^ However, human sensitivity has been reported to increase with vibrotactile stimulation above 100 Hz and decrease above 320 Hz; the optimum stimulation appears to be at 250 Hz,^31^ and there is a greater response to a 300-Hz tactile stimulus than to a 30-Hz stimulus.^32^ These results suggest that a somatotopic map of BA 3 can be developed by using not only flutter stimulation but also vibration stimulation. Because most humans are capable of distinguishing high-frequency vibration in each phalanx of a finger, intradigit mapping with high-frequency vibrations seems to be a feasible approach despite the disadvantage of wide receptive fields.

The aim of this study was to build an inter-/intradigit somatotopic map of BA 3 in response to high-frequency vibration stimulation and compare it with the flutter stimulation-based somatotopic map. Furthermore, we aimed to investigate inter-/intradigit distances as well as inter- and intradigit distances in the somatotopic distribution of BA 3 to comprehend the characteristics of finger somatotopy. We applied 250-Hz high-frequency vibration stimulation on 3 phalanxes (distal [p1], intermediate [p2], and proximal [p3]) of 4 different fingers (index, middle, ring, and little) of human subjects to generate a somatotopic map based on the peak coordinates in BA 3. The interdigit, intradigit, and inter-/intradigit distances (Euclidean distances) were calculated and interpreted accordingly.

## METHODS

### Participants

Ten healthy male (26.6 ± 2.52 years’ old) college students participated in the study. All subjects were right-handed as evaluated by the revised Edinburgh test.^33^ None of the participants reported having a history of psychiatric, physiological, or neurological disorders. The overall procedure was explained to all participants, who in turn gave their consent for the procedure. Written informed consent was obtained from all participants before the experiment. All experimental procedures were approved by and performed under the regulations of the Institutional Review Committee of Korea University (KU-IRB-11-46-A-1).

### Vibrotactile Stimulation

An MR-compatible vibrotactile stimulator using a planar-coil-type actuator was developed for generating vibrations at the target frequency.^34^ The planar-coil-type actuator consisted of electric wires in a planar coil form and generated vibrations within a static magnetic field of a magnetic resonance (MR) scanner. Stimulus parameters, including the vibration frequency (250 Hz), were controlled with E-Prime software (Psychology Software Tools, Inc. Sharpsburg, PA, USA) and synchronized by a trigger signal sent through a parallel port. Each stimulator weighed 11 g and had a contact surface area of 1 × 1 cm^2^.

### Experimental Design

**FIGURE 1:**
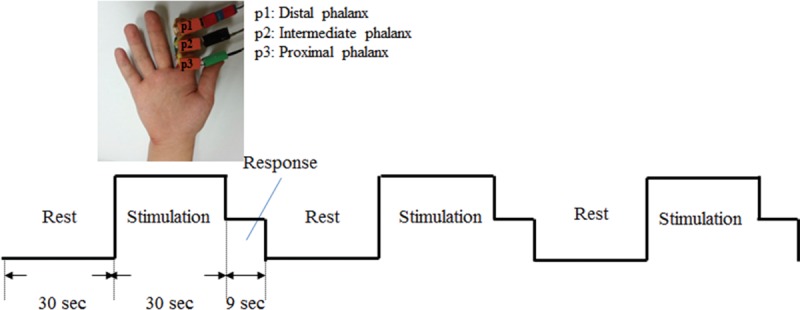
Image of the actual stimulator attached and description of experimental design. Subjects remained resting without any stimulation (rest phase), received vibrotactile stimulation on a random phalanx (stimulation phase), and responded with regard to which phalanx was stimulated by pressing a button in the other hand (response phase).

The experiment was performed on the right hand of all participants, who kept their eyes closed and wore a headset during the whole experiment (or phase) to minimize the effect of auditory and visual distractions. Three vibrotactile stimulators were attached to each phalanx (p1, p2, and p3) of a selected finger in a single session (Figure 1). A single fMRI trial consisted of 3 phases: rest (30 s), stimulation (30 s), and response (9 s). After the participants rested with their eyes closed for 30 s during the rest phase, vibration stimulation was applied to a random phalanx (p1, p2, or p3) for 30 s during the stimulation phase. For the purpose of confirmation, participants were asked to respond during a consecutive 9-s response phase with regard to which phalanx felt the vibration by pressing a response button with their left hand. Because a single session consisted of 3 trials, all 3 phalanxes were experimented upon in a counterbalanced random order. Each session was repeated 3 times for each finger (index, middle, ring, and little finger) resulting in 12 experimental sessions in total (3 sessions/finger × 4 fingers = 12 sessions). All sessions were interspaced with 5 minutes of resting time. Only the data from 8 (25.6 ± 2.92 years’ old) of 10 subjects who responded to the stimulation with 100% accuracy were used for further fMRI data analysis.

### MR Image Acquisition

We obtained brain images by using a 3-tesla MRI system (Magnetom TrioTim, Siemens Medical Systems, Erlangen, Germany) equipped with a standard head coil (32-channel). First, we acquired anatomical brain images by using a T1-weighted 3D MPRAGE sequence. The parameters of the anatomical image acquisition were as follows: a repetition time (TR) of 1900 ms, echo time (TE) of 2.48 ms, flip angle of 9 degree, field of view (FOV) of 200 mm, and spatial resolution of 0.8 × 0.8 × 1 mm^3^. Next, we acquired functional brain images with a T2∗-weighted gradient echo-planar imaging (EPI) sequence. The parameters of the functional image acquisition were as follows: a TR of 3000 ms, TE of 30 ms, flip angle of 0 degree, FOV of 192 mm, slice thickness of 2 mm, and in-plane resolution of 1.5 × 1.5 mm^2^ (refer to our previous study^35^ for the MR image acquisition).

### fMRI Data Analysis

The fMRI data were analyzed by using the Statistical Parametric Mapping 8 software package (SPM8; Wellcome Department of Cognitive Neurology, London, UK). All functional images were realigned to correct for head movements by using affine transformation routines built into SPM 8. The realigned scans were co-registered to the participant's anatomic images that were obtained within each session. The anatomical image was then segmented into white matter, gray matter, and cerebrospinal fluid. The mean EPI image of each subject was directly warped into the standard EPI template (Montreal Neurologic Institute) during the normalization step. The time-series data were motion-corrected by Sinc interpolation and filtered with a 240-s high-pass filter to remove artifacts owing to cardiorespiratory and other cyclical influences. The functional images were then smoothed with a 3-mm full-width-half-maximum (FWHM) isotropic Gaussian kernel before statistical analyses. Although the size of the isotropic Gaussian kernel used in our previous studies was 6 or 8 mm,^36^ this was scaled down to 3 mm in the present study. Our aim was to differentiate cortical areas in response to stimulations of individual digits and phalanx within a local brain region (eg, BA 3). The statistical analysis was conducted both individually and as a group by using the general linear model and the theory of Gaussian random fields with SPM 8. Statistical parametric maps were computed with t-statistics. Individual subjects were analyzed at a significance threshold of *P* < 0.05, which was corrected by using the topological peak-false discovery rate (FDR). We used random effect analysis to compute group activation^37^ (see our previous study^36^ for the overall data analysis procedure).

Activation in BA 3 extracted with the region of interest (ROI) analysis was baseline-corrected with the subtraction method (stimulation phase – rest phase) to yield peak coordinates for each finger–phalanx combination. The location of BA3 was defined using the anatomical criteria at both the subject and group analysis levels. The peak coordinates in BA 3 were defined as the location of a voxel with the highest activation intensity measured by *t* value, represented in the MNI coordinate system (x-, y-, and z-axes). The peak coordinates were mapped at 12 locations (3 phalanxes × 4 fingers) for each participant and for the group.

### Measure of Inter-, Intra-, and Inter-/Intradigit Distances

Interdigit, intradigit, and inter-/intradigit somatotopic distances were calculated for all finger–phalanx combinations by using difference vectors and the Euclidean distance (ED) of peak coordinates.^4,6,13,15,17^

**FIGURE 2:**
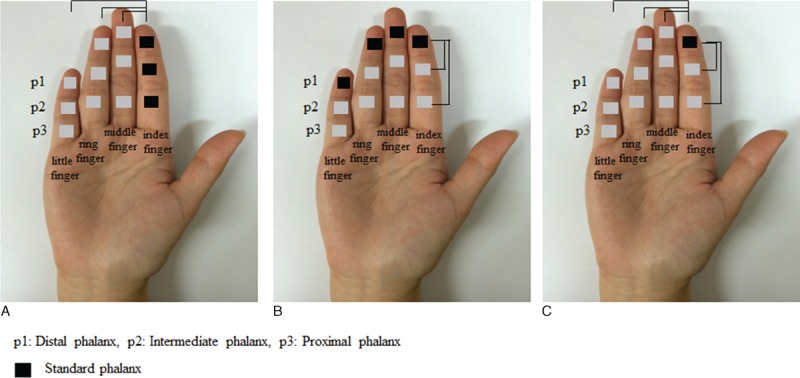
Definition of the stimulated locations. (A) The interdigit distance was calculated as the distance between peak coordinates in BA 3 of each phalanx of the index finger (p1, p2, or p3 in black square) and the corresponding phalanx of other fingers (middle, ring, and little). (B) The intradigit distance was calculated as the distance between p1 (black square) and others (p2, and p3) within each finger. (c) The inter-/intradigit distance was calculated as the distance from p1 of the index finger (black square) to other finger–phalanx combinations.

First, the interdigit distances were calculated from the difference between peak coordinates of the index finger (p1–p3) and the corresponding phalanxes of other fingers (Figure 2A). For example, the distance between the first phalanxes was extracted from difference vectors of the middle finger p1, ring finger p1, and little finger p1 with respect to the index finger p1 (standard phalanx) in the MNI coordinate system (Eq. 1(a)). The interdigit differences of phalanxes p2 and p3 were calculated in the same manner. 


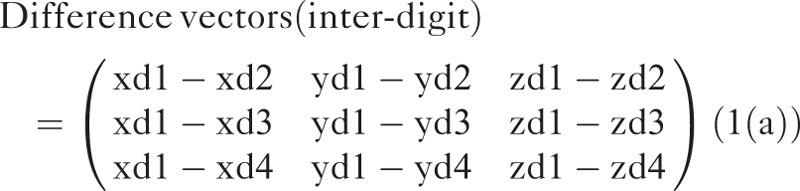


Here, xd, yd, and zd denote coordinates along the x-, y-, and z-axes, respectively. The numbers in the notations denote different fingers: 1, index; 2, middle; 3, ring; and 4, little.

Second, the intradigit distance of phalanxes was generated within each finger with respect to p1 (standard phalanx). For instance, distances within the index finger were extracted from difference vectors between p1 and p2 as well as p1 and p3. Intradigit differences of the middle, ring, and little fingers were also calculated in the same manner (Eq. 1(b)). 


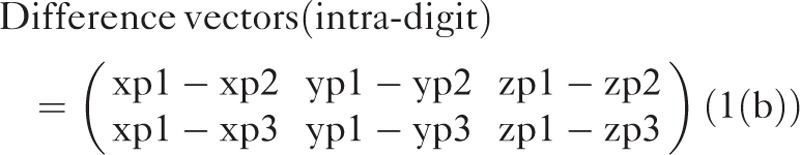


Here, xp, yp, and zp denote coordinates along the x-, y-, and z-axes, respectively. The numbers in each notation denote different phalanxes within a finger: 1, distal; 2, intermediate; and 3, proximal.

Third, for the inter-/intradigit distance, difference vectors of all other combinations were generated with respect to peak coordinates of the standard phalanx index p1 (Figure 2; see Eq. 1(c)). Thus, the vector contains 11 rows for all of the differences and 3 columns for 3-dimensional axes (x-, y-, and z-axes). 


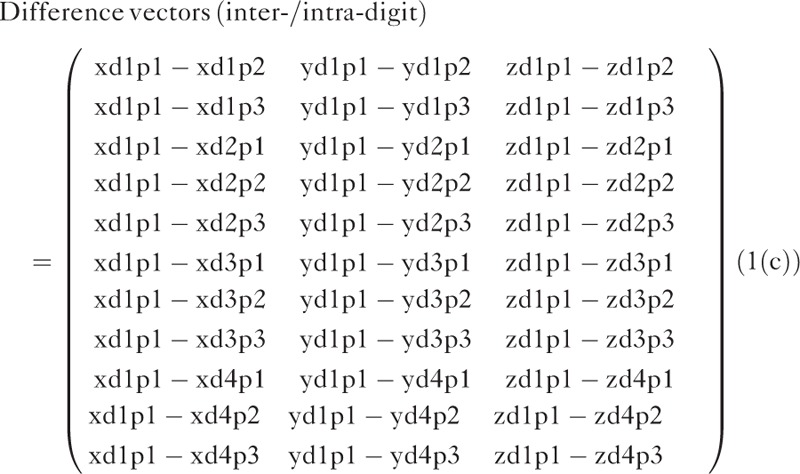


Here, the first 3 letters (xd1–xd4) denote the fingers from index to little, and the latter 2 letters (p1–p3) denotes phalanxes from distal to proximal.

The Euclidean distance of the somatotopic peak coordinates was then calculated as the rooted sum of squares of every row component in the difference vector. For example, Eq. 2 presents the intradigit distance between p1 and p2 (the first row of Eq. 1(b)). All rows of Eqs. 1 (a)-(c) were processed accordingly to generate inter-, intra-, and inter-/intradigit distances, respectively. 


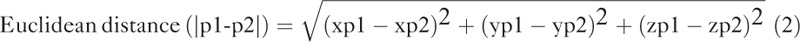


We performed the Friedman test (PASW 18.0) to validate the statistical significance of the inter-, intra-, and inter-/intradigit difference vectors and Euclidean distances. Then, we used the Wilcoxon matched paired *t* test (PASW 18.0) for multiple-comparison correction. For the interdigit distance, we evaluated distances between p1 of each finger and tested whether the distances were statistically different by using the aforementioned methods. The same procedure was repeated to test the interdigit distances for p2 and p3. For the intradigit distance, we evaluated distances between the phalanxes for each finger and tested whether the distances were statistically different by using the same method. For the inter-/intradigit distance, we evaluated the distances of every phalanx of every finger relative to p1 of the index finger and tested the statistical differences of the distances.

## RESULTS

Eight of the 10 participants exhibited 100% accuracy during the response phase by discriminating the exact phalanx stimulated (94.4% and 97.2% for the nonperfect responders). This indicates that the high-frequency vibrotactile stimulation used in this study could be distinctly perceived for both the inter- and intra-digit aspects. The remaining 2 participants who showed a behavioral discrimination accuracy of <100% were excluded from the neural data analysis because this may indicate that they could not perceive a stimulus and/or the location of a stimulus for some stimulus presentation, and the corresponding neural responses may not directly contain the information of the stimulus.

**FIGURE 3:**
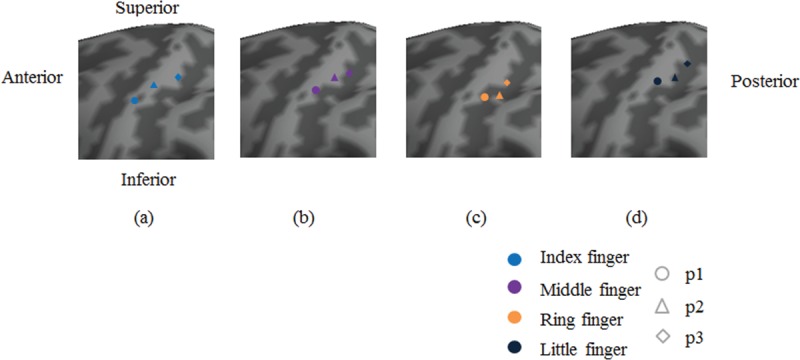
Visualized group analysis results of BA 3 activation peak coordinates (red points).

**FIGURE 4:**
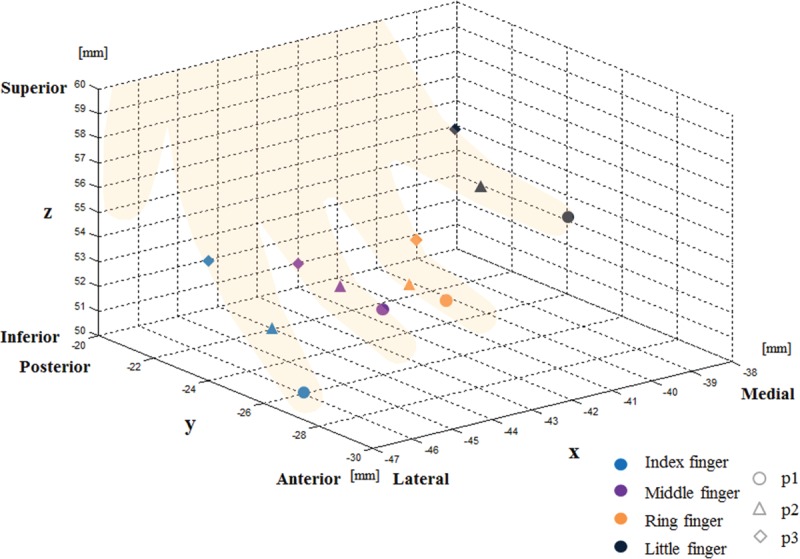
250-Hz vibraotactile stimulation-evoked BA 3 peak coordinates represented in MNI coordinates (mm) of lateral-medial (x-axis), anterior-posterior (y-axis), and inferior-superior (z-axis) 3-dimensional axes. Whereas the index to little fingers consecutively presented peak coordinates directed along the lateral-to-medial axis, phalanxes p1–p3 for all fingers were aligned along the anterior-to-posterior and inferior-to-superior axes.

Table 1 (MNI coordinates) and Figure 3 describe the group analysis results of the peak coordinates, whereas Figure 4 represents the results along the 3-dimensional x- (lateral–medial), y- (anterior–posterior), and z-axes (inferior–superior). Figure 4 maps the peak coordinates from the index finger to the little finger in the directions of the lateral-to-medial and inferior-to-superior axes in BA 3. For p2 and p3, the directions of the finger coordinates could only be distinguished along the lateral-to-medial axis. Intradigit mappings were directed along the anterior-to-posterior and inferior-to-superior axes for all 4 fingers.

**TABLE 1:**
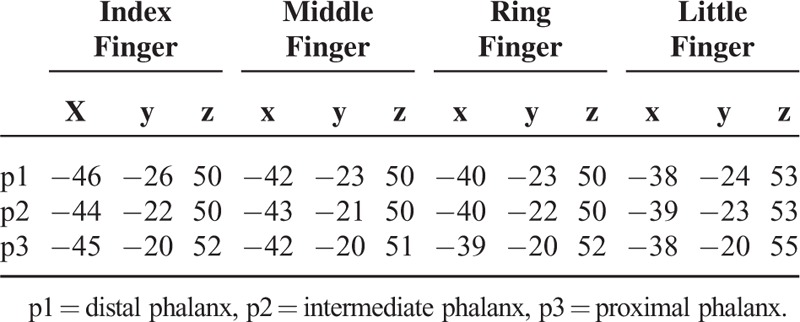
Group Analysis Results of the BA 3 Activation Peak Coordinates (Anatomical Criteria) in MNI Coordinates (mm) During 250-Hz Vibrotactile Stimulation on Each Phalanx of the 4 Fingers

Table 2 presents the mean and standard deviation of the inter-, intra-, and inter-/intradigit difference vectors and Euclidean distances from the standard phalanx of the eight participants who distinguished the location of stimulus perfectly. The number 0 represents the standard phalanx. Although the distance vector itself exhibited no statistical significance, there were observable differences in the Euclidean distance. The Friedman test revealed significant interdigit distances for p1 (*P* = 0.044) as well as p2 (*P* = 0.038). Significant intradigit distances were revealed for the index finger (*P* = 0.048), ring finger (*P* = 0.032), and little finger (*P* = 0.029). There was a significant inter-/intradigit distance across the phalanxes and fingers (*P* = 0.045).

**TABLE 2:**
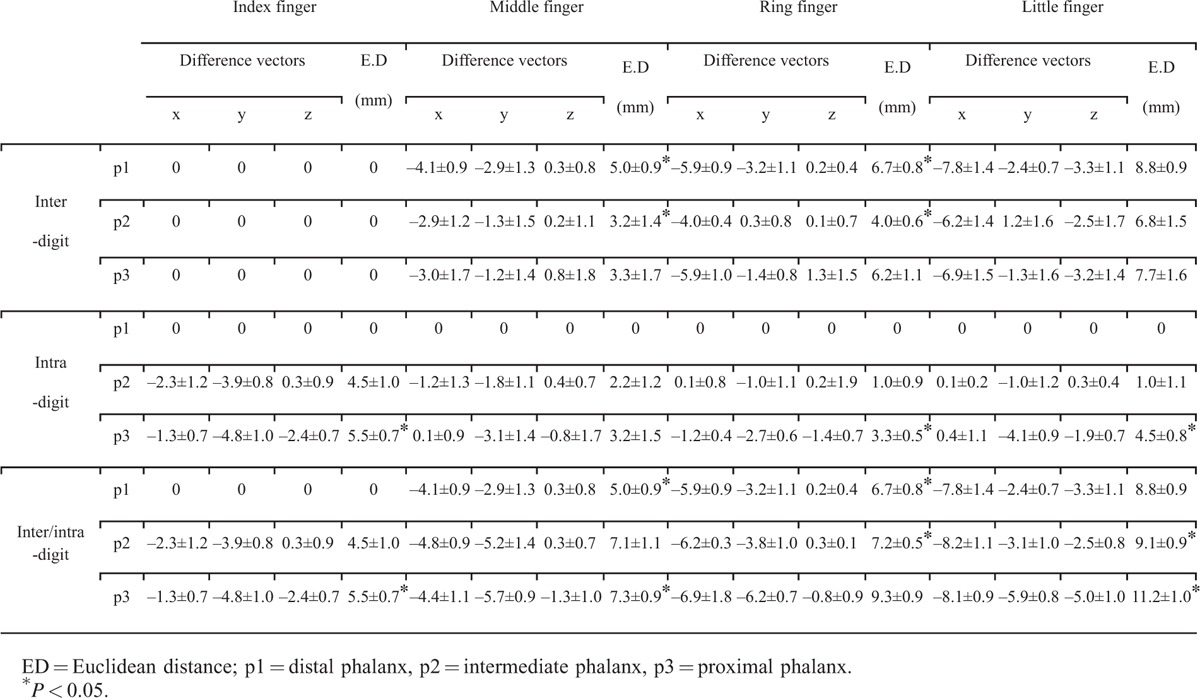
Interdigit, Intradigit, and Inter-/Intradigit Distances Between BA 3 Peak Coordinates Represented as Difference Vectors and Euclidean Distances (mean ± SD Through Subject Analysis)

Statistically significant interdigit Euclidean distances were as follows: for the distal phalanx, 5.0 ± 0.9 mm (*P* = 0.049) between index p1 and middle p1 and 6.7 ± 0.8 mm (*P* = 0.026) between index p1 and ring p1; for the middle phalanx, 3.2 ± 1.4 mm (*P* = 0.037) between index p2 and middle p2 and 4.0 ± 0.6 mm (*P* = 0.050) between index p2 and ring p2. None of the distances between fingers in the proximal phalanx (p3) passed the significance criterion of *P* < 0.05. In terms of intradigit distances, the significance threshold was passed for the following: 5.5 ± 0.7 mm (*P* = 0.035) for p1 and p3 of the index finger, 3.3 ± 0.5 mm (*P* = 0.040) for p1 and p3 of the ring finger, and 4.5 ± 0.8 mm (*P* = 0.032) for p1 and p3 of the little finger.

Inter-/intradigit distances were comprehensively calculated with respect to the peak coordinates of the distal phalanx of the index finger. The statistically significant distances were as follows: 5.5 ± 0.7 mm (*P* = 0.035) to index p3, 5.0 ± 0.9 mm (*P* = 0.049) to middle p1, 7.3 ± 0.9 mm (*P* = 0.037) to middle p3, 6.7 ± 0.8 mm (*P* = 0.026) to ring p1, 7.2 ± 0.5 mm (*P* = 0.028) to ring p2, 9.1 ± 0.9 mm (*P* = 0.036) to little p2, and 11.2 ± 1.0 mm (*P* = 0.049) to little p3.

## DISCUSSION

### Interdigit Somatotopic Map and Distance

Previous studies with piezoelectric, brush, or electric stimulation on the distal phalanx of 5 different fingers have reported that somatotopic coordinates in S1 from the thumb to the little finger were directed along the lateral-to-medial, inferior-to-superior, and anterior-to-posterior axes.^6,13,16,17^ Our study on the rather unexplored high-frequency vibrotactile stimulation of 250 Hz showed similar tendencies. The coordinates from index p1 to little p1 in BA 3 were directed along the lateral-to-medial and inferior-to-superior axes, but the tendency along the anterior-to-posterior axis was unclear.

Our results also shed some light on the inter-digit distances between phalanxes apart from the distal one. The peak coordinates of the intermediate (p2) and proximal (p3) phalanxes of the 4 fingers were also aligned along the lateral-to-medial axis. However, the tendency toward other axes was less clear in these cases.

Nelson and Chen^6^ experimented on using 23-Hz piezoelectric stimulation on the distal phalanx of 5 different fingers. The somatotopic distances from the thumb peak coordinate were 7.2, 11.2, 16, and 17 mm to the index, middle, ring, and little fingers, respectively.^6^ In the same manner, other studies that used electrical stimulation on the first phalanx of the 5 fingers have reported the following somatotopic distances: 5.7 mm between the thumb and index finger, 5.4 mm between the index and middle fingers, 4.7 mm between the middle and ring fingers, 3.7 mm between the ring and little fingers, and 15.5 mm between the thumb and little fingers.^4,17^ The distances between the thumb and little finger (17.2 mm) and between the thumb and index finger (10.6 mm) were reported in a brush-stimulated experiment.^13^ In sum, even though the results from the above references were acquired with different stimulation methods, the interdigit distance between the thumb and little finger can be considered to be around 15.5 to 17.2 mm, whereas the distance between the thumb and index finger is 5.7 to 10.6 mm. Although a direct comparison would be difficult because we chose not to test the thumb based on the findings that the activation of the finger is most pronounced in BA 1 and BA 2,^6,8^ a rather indirect comparison by inference is possible. Inferences from the results of Nelson and Chen^6^ with an assumed linear arrangement for the index-to-other fingers (4.0, 8.8, and 9.8 mm) are quite close to the corresponding results from our study (index–middle: 5.0 mm, index–ring: 6.7 mm, index–little: 8.8 mm).

The interdigit distances and their statistical significance tended to be greater in p1 compared to p2 or p3. This phenomenon is likely to be caused by the greater perceived stimulus intensity associated with the denser distribution of cutaneous mechanoreceptors in the distal phalanx.^15,29,30^

The direction of the somatotopic coordinates for p1 and their distances did not exhibit much discrepancy in terms of the vibrated stimulation, which our study was based on, and flutter stimulation, which most preceding studies were based on. Our results did not reveal whether the digit somatotopic maps for vibration and flutter stimulations are anatomically identical. However, they may indicate that the digit tactile location information transmitted through different sensory afferents from vibration and flutter stimulations is represented by a shared structure in BA 3.^38^ Further experiments and analyses will be needed in this regard.

### Intradigit Somatotopic Map and Distance

Intradigit somatotopic mapping has been of less interest compared to numerous interdigit studies.^4,6,13,16,17^ Schweisfurth et al^15^ tested 32 Hz piezoelectric stimulation on the distal phalanx (p1) and a base part (p4) in the index and little fingers. They reported that the somatotopic BA 3 peak coordinates of p1 to p4 in the little finger were along the anterior-to-posterior direction.^15^ Sanchez-Panchuelo et al^26,27^ provided 30-Hz flutter and 150-Hz vibration stimuli to each of 3 phalanxes of the index finger and reported an intradigit somatotopic map in BA 3 showing an anterior-to-posterior direction for the first-to-third phalanx. Schweisfurth et al^28^ provided a 32-Hz flutter stimulus to each of the 3 phalanxes of all 5 fingers and reported an intradigit somatotopic map for individual fingers in BA 3 that showed anterior-to-posterior and lateral-to-medial directions for the order from p1 to p3. However, they only focused on intradigit somatotopic maps and did not unify inter- and intradigit somatotopic maps at the same time. Blankenburg et al^12^ reported that electrical stimulation on each phalanx in the middle finger yielded somatotopic coordinates in the same direction in the order from distal to proximal phalanxes.^12^ Overduin and Servos^14^ experimented with air stimulation on the thumb, index, and ring fingers at 1.5-cm intervals but failed to develop an intradigit somatotopic map.^14^ In our study, somatotopic coordinates of phalanxes from p1 to p3 appeared to be aligned in the anterior-to-posterior direction, which is in line with previous findings. In addition, we observed somatotopic coordinates from p1 to p3 in the inferior-to-superior direction.

Schweisfurth et al^28^ reported intradigit distances in BA 3 for individual fingers from the somatotopic map generated by 32-Hz flutter stimulation. The resulting distances were as follows: (index finger) 4.0 mm for p1 to p2 and 6.2 mm for p1 to p3; (middle finger) 2.9 mm for p1 to p2 and 6.6 mm for p1 to p3; (ring finger) 2.9 mm for p1 to p2 and 5.0 mm for p1 to p3; (little finger) 2.3 mm for p1 to p2 and 3.4 mm for p1 to p3. Our study presented significant intradigit distances as follows: 5.5 mm for p1 to p3 of the index finger, 3.3 mm for p1 to p3 of the ring finger, and 4.5 mm for p1 to p3 of the little finger. These results are similar to those of Schweisfurth et al^28^, which suggest consistent intradigit distances in BA 3. Slight differences in the distances may be caused by the different stimulus types (flutter versus vibration).

The high-frequency vibrotactile stimulus used here (ie, vibration) is more likely to stimulate the Pacinian corpuscle than the Meissner corpuscle. Although the Pacinian corpuscle is known to possess a large receptive field that covers the entire finger,^29,30^ our results suggest possible intradigit discrepancies for the BA 3 finger somatotopy in response to high-frequency vibration. We surmise that, even though stimulation on a specific phalanx can cause responses from multiple Pacinian corpuscles with overlapped receptive fields, there may exist differences in response patterns (intensity and/or latency) depending on their proximity. Therefore, those differences sent through sensory afferents may enable cortical networks to specify the location of the stimulation. However, further neurophysiological studies would be required to uncover the underlying mechanisms in depth.

### Inter-/Intradigit Somatotopic Map and Distance

The inter-/intradigit somatotopic map revealed a lateral-to-medial trend for fingers (in the order of index, middle, ring, and little fingers) as well as an anterior-to-posterior trend for phalanxes (from p1 to p3 consecutively) of peak activation coordinates in BA 3. This finding provides an integrated view into the results from previous inter- and intradigit studies conducted separately.

**FIGURE 5:**
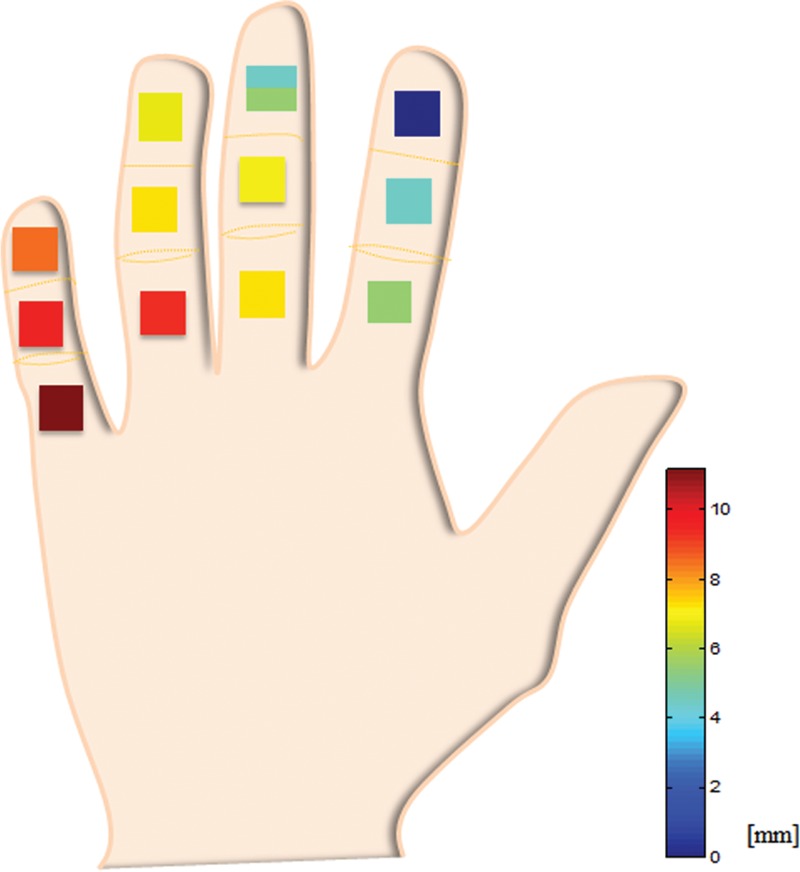
Inter-/intradigit distance calculated with respect to p1 of the index finger. The mapped locations appeared to propagate in a radial form: the most proximal group (index p2, middle p1, middle p2) followed by the second-most (ring p1, middle p2, ring p2, middle p3), third-most (little p1, little p2, ring p3), and final group (little p3). In line with the previous report, the distance was greatest between index p1 and little p3.

In our study, inter-/intradigit distances were calculated with respect to p1 of the index finger (Table 2). Generally, the absolute magnitude of the interdigit distance was greater than that of the intradigit distances. The somatotopic distance was the greatest between p1 of the index finger and p3 of the little finger. This coincides with the report of Schweisfurth et al^15^, who found that activation in those 2 locations presented a larger cortical representation and was more pronounced than for other finger–phalanx combinations. Moreover, as illustrated in Figure 5, the proximity of finger–phalanx combinations appeared to propagate radially from p1 of the index finger. In other words, index p2, middle p1, and middle p2 formed the most proximal group, followed by the next group (ring p1, middle p2, ring p2, middle p3), third group (little p1, little p2, ring p3), and fourth group (little p3).

### The Applications of High-Frequency Vibrotactile Stimulation

The high-frequency (250 Hz) vibrotactile stimulation used in our study can be implicated in a wide range of applications. A number of studies have demonstrated the improvement of human performance such as balance and postural stability using the stochastic resonance (SR) effect,^39^ which refers to a phenomenon wherein a nonlinear system becomes more sensitive to a small external input with an intermediate level of noise.^40^ A recent study by Trenado et al has revealed that the SR effect on human sensorimotor performance is greater with broadband white noise (0–300 Hz) and high-frequency noise (250–300 Hz) than with low-frequency noise (0–5 Hz), as well as more pleasant tactile sensations induced by broadband or high-frequency noise than by low-frequency noise.^39^ One may be able to utilize this increased SR effect with high-frequency vibrotactile stimulation for a variety of applications, including intelligent haptic interfaces,^41^ SR-based therapies for balance and position,^42^ rehabilitation of somatosensory functions lost by age or diseases, to name a few. Also, our results of inter-/intradigit somatotopic maps with high-frequency vibrotactile stimulation can be used together with the SR-based therapy for rehabilitation of hand and fingers and monitoring cortical plastic changes in patients with stroke or focal hand dystonia.

## LIMITATIONS

The present study provided a vibration stimulation of 250 Hz to each of 3 phalanxes of 4 fingers to develop an inter-/intradigit somatotopic map in BA 3 from SI. Previous studies provided flutter stimulation to fingers and reported interdigit maps in BA 1, BA 2, and BA 3.^4,6,8,13,16,17^ We also analyzed activations in BA 1 and BA 2 along with BA 3, but found significant activations in only 2 or 3 people out of all participants. This means that BA 3 may be a dominant area that receives tactile sensory input and represents robust inter-, intra- and inter-/intradigit somatotopy, whereas BA 1 and BA 2 are relatively inconsistent in that matter. We speculate that a larger number of subjects may be required to clarify differences between BA 1, 2, and 3 for inter-/intradigit somatotopy and that high-frequency vibration may evoke less activation in BA 1 and BA 2 compared with BA 3. Further studies are needed to explore this.

## CONCLUSIONS

We applied high-frequency (250 Hz) vibrotactile stimulation on 3 phalanxes (p1–p3) in each of the 4 fingers (index, middle, ring, and little) to investigate the corresponding somatosensory cortical activity, particularly in BA 3. We developed an inter-/intradigit somatotopic map for high-frequency vibration stimulation that forms a similar distribution to those generated from flutter (low-frequency) stimulation. The inter- and intradigit distances of high-frequency vibration were on par with previous findings drawn from other stimulation modalities such as low-frequency piezoelectric, brush, air, or electric stimulation. The comprehensive inter-/intradigit somatotopic mapping obtained in this study is expected to contribute toward generating an integrated model for comprehensive and precise somatotopic mapping of fingers and their components.
